# Cognitive Behavioral Therapy Program for Cannabis Use Cessation in First-Episode Psychosis Patients: A 1-Year Randomized Controlled Trial

**DOI:** 10.3390/ijerph19127325

**Published:** 2022-06-15

**Authors:** Itxaso González-Ortega, Enrique Echeburúa, Susana Alberich, Miguel Bernardo, Eduard Vieta, Gonzalo Salazar de Pablo, Ana González-Pinto

**Affiliations:** 1 Centre for Biomedical Research in the Mental Health Network (CIBERSAM), 28029 Madrid, Spain; enrique.echeburua@ehu.eus (E.E.); susana.alberichmesa@osakidetza.eus (S.A.); bernardo@clinic.cat (M.B.); evieta@clinic.cat (E.V.); anamaria.gonzalez-pintoarrillaga@osakidetza.eus (A.G.-P.); 2 Bioaraba Research Institute, Department of Psychiatry, Araba University Hospital, 01004 Vitoria, Spain; 3 Department of Personality, Assessment and Psychological Treatment, National University of Distance Education (UNED), 01008 Vitoria, Spain; 4 Department of Personality, Assessment and Psychological Treatment, University of the Basque Country, Biodonostia, 20018 San Sebastian, Spain; 5 Department of Fundamental Mathematics, National University of Distance Education (UNED), 01008 Vitoria, Spain; 6 Department of Psychiatry, Hospital Clinic of Barcelona, Neuroscience Institute, University of Barcelona, 08036 Barcelona, Spain; 7 Institut d’Investigacions Biomèdiques August Pi i Sunyer (IDIBAPS), 08036 Barcelona, Spain; 8 Department of Child and Adolescent Psychiatry, Institute of Psychiatry, Psychology & Neuroscience, King’s College London, London SE5 8AB, UK; gonzalo.salazar_de_pablo@kcl.ac.uk; 9 Early Psychosis: Interventions and Clinical-detection (EPIC) Lab, Department of Psychosis Studies, Institute of Psychiatry, Psychology & Neuroscience, King’s College London, London SE5 8AB, UK; 10 Child and Adolescent Mental Health Services, South London & Maudsley NHS Trust, London SE11 6JJ, UK; 11 Department of Neurosciences, University of the Basque Country, 48940 Leioa, Spain

**Keywords:** first-episode psychosis, cannabis use, cognitive behavioral therapy, outcome, randomized controlled trial

## Abstract

Despite the negative influence of cannabis use on the development and prognosis of first-episode psychosis (FEP), there is little evidence on effective specific interventions for cannabis use cessation in FEP. The aim of this study was to compare the efficacy of a specific cognitive behavioral therapy (CBT) for cannabis cessation (CBT-CC) with treatment as usual (TAU) in FEP cannabis users. In this single-blind, 1-year randomized controlled trial, 65 participants were randomly assigned to CBT-CC or TAU. The primary outcome was the reduction in cannabis use severity. The CBT-CC group had a greater decrease in cannabis use severity and positive psychotic symptoms over time, and a greater improvement in functioning at post-treatment than TAU. The treatment response was also faster in the CBT-CC group, reducing cannabis use, anxiety, positive and general psychotic symptoms, and improving functioning earlier than TAU in the follow-up. Moreover, patients who stopped and/or reduced cannabis use during the follow-up, decreased psychotic symptoms and increased awareness of disease compared to those who continued using cannabis. Early intervention based on a specific CBT for cannabis cessation, may be effective in reducing cannabis use severity, in addition to improving clinical and functional outcomes of FEP cannabis users.

## 1. Introduction

Cannabis is the most commonly used substance in first-episode psychosis patients (FEP) with a cannabis use rate of 64% among these patients, of which 30% have a cannabis use disorder [1]. Meanwhile, the prevalence of problematic cannabis use among the young in the general population is between 1.5 and 2.9% [2]. There is evidence about cannabis use as a risk factor in the development and evolution of psychosis [3]. Specifically, cannabis use is associated with an earlier psychosis onset [4,5,6,7] and increased risk of transition in individuals at clinical high risk of psychosis [7]. The early age of onset of cannabis use and the severity and frequency of use was also associated with an increased risk of developing psychosis [4,7,8,9,10], indicating a dose-dependent response relationship [9,11]. Moreover, the relationship between cannabis use and psychosis may be modulated by sex [12] and genetic factors, increasing the risk in individuals with genetic vulnerability [7,13,14].

Cannabis use also has a negative impact on the clinical and functional outcomes of FEP patients. Its consumption has been associated with poor adherence to psychological and pharmacological treatment [15,16,17], increased severity of psychotic symptoms [11,18,19], and a greater risk of relapse and hospitalizations [20,21,22] in these patients. Moreover, patients with FEP who use cannabis have a poorer functional outcome at follow-up [18,19,23,24,25,26]. Cannabis use cessation, conversely, has been related to an improvement in clinical and functional outcomes and a lower risk of relapse in FEP [18,20,21]. Cannabis use, therefore, should be a priority objective in the treatment of early psychosis in order to reduce cannabis consumption and improve outcomes in FEP. However, despite the negative influence of cannabis use on the development and prognosis of the disease, there is little evidence with regard to effective specific interventions to reduce cannabis in FEP. 

The randomized controlled trial (RCT) studies that have assessed the effectiveness of psychosocial interventions to reduce cannabis use in FEP included interventions based on motivational intervention [27] or combined interventions based on motivational intervention and cognitive behavior therapy [28,29,30,31]. These studies found no benefits from the interventions compared with treatment as usual condition (TAU) in terms of reducing cannabis, except in the study conducted by Bonsack et al. [27], although this benefit was not sustained at follow-up. There was also no improvement in clinical and functional outcomes; only in one study did patient quality of life improve at post-treatment [30]. Generally, the results of these studies failed to draw definite conclusions and clearly indicate whether the interventions were effective in terms of reducing cannabis use, including the tendencies towards reductions in the amount but not the frequency of cannabis use [27,29,31]. A recent RCT conducted by Cather et al. [32], assessed the efficacy of an integrated program composed of family psychoeducation and individual resiliency training for substance use in FEP patients and found no reduction in substance use at follow-up compared to TAU, suggesting that modifications to the program are needed, with special emphasis on earlier intervention [33]. 

Since there is clear evidence about the association between cannabis use and worse clinical and functional outcomes of FEP and since RCTs have not found an improvement in outcomes for these patients, further research is needed to determine the most effective strategies for addressing cannabis use in FEP patients. For this reason, we designed a psychotherapy for FEP cannabis users based not only on the cannabis use reduction but also on psychosis prevention, with a dual approach. 

The main aim of this article was to compare the efficacy of a specific cognitive behavioral therapy (CBT) program for cannabis cessation with standard treatment in patients with FEP who are cannabis users. The specific objectives of the study were:

1. To assess whether a specific CBT program for cannabis cessation is associated with a greater reduction in the use of cannabis than standard treatment at post-treatment and in the follow-up.
2. To assess whether this type of program for cannabis cessation is associated with better outcomes of the psychotic disorder (i.e., reduction in symptoms and improvement in psychosocial functioning) than standard treatment at post-treatment and in the follow-up.
3. To analyze the relation between cannabis abstinence and clinical and functional outcomes of patients.

## 2. Materials and Methods 

### 2.1. Design 

**Figure 1:**
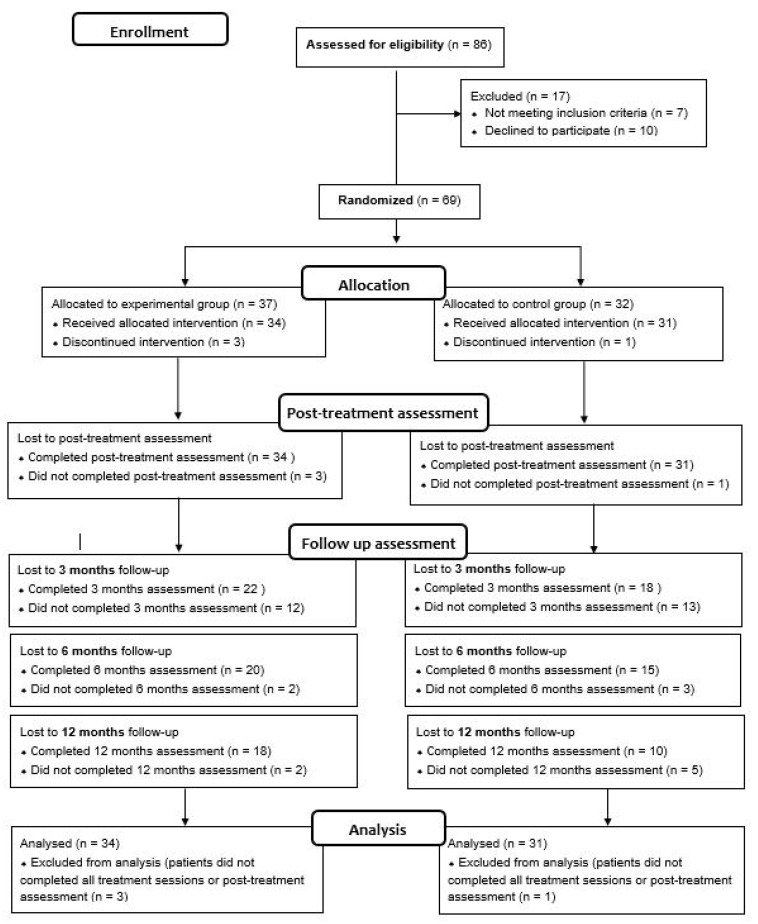
The CONSORT flow diagram.

This is a single-blind RCT registered in 2014 (ClinicalTrials.gov, Identifier NCT02319746). The study protocol is described in a previous article conducted by González-Ortega et al. [34]. This RCT fulfills the CONSORT (CONsolidated Standards of Reporting Trials) guidelines, checklist, and flow diagram (Figure 1).

The study was carried out in accordance with the Helsinki Declaration of 1975 and was approved by the Clinical Research Ethics Committees of Araba University Hospital (HS/EC/2012-003) and the Clinic Hospital of Barcelona (HCB/2016/0639). 

### 2.2. Participants

The study was conducted on FEP patients who were cannabis users recruited between 2013 and 2019 from Araba University Hospital and Clinic Hospital of Barcelona. 

The sample size calculation was performed using Ene 2.0 software and based on previous studies related to this population [27,28,29,30,31]. To achieve an 80% power to detect mean differences from the null hypothesis, H0: μ1 = μ2, using a bilateral Student’s *t*-test for two independent samples, with a significance level of 5%, an enrollment of 30 patients for each group was estimated, meaning a total of 60 patients for the study.

#### 2.2.1. Inclusion Criteria

The study inclusion criteria for patients were:

1. Being diagnosed as FEP according to the revised fourth edition of the Diagnostic and Statistical Manual of Mental Disorders, Fourth Edition, Text Revision (DSM-IV-TR) [35] (i.e., schizophreniform disorder, schizoaffective disorder, delusional disorder, bipolar disorder with psychotic symptoms, atypical psychosis, brief psychotic disorder, non-specified psychotic disorder, or major depressive disorder with psychotic symptoms).
2. Meeting dependence or abuse of cannabis criteria according to the Diagnostic and Statistical Manual of Mental Disorders, Fourth Edition, Text Revision (DSM-IVTR) [35] and the scores of the European Addiction (Europ-ASI) [36,37] (scores of 4 to 7: abuse; scores of 8 to 9: dependence) (Table 1).
3. Aged between 15 and 40 years. In the case of minors (under 18 years of age), written informed consent was requested from their parents or guardians.

#### 2.2.2. Exclusion Criteria

The study exclusion criteria for patients included organic brain pathology and/or mental disability according to DSM-IVTR criteria. 

### 2.3. Procedure

Patients, who met inclusion criteria and signed the informed consent to participate in the study, were assessed and randomly assigned to one of the treatment groups by permuted block randomization with a block size of 4 and a 1:1 allocation using a computer-generated random sequence. The allocation sequence was prepared by an independent person not otherwise involved in the clinical trial. 

All patients were assessed at baseline, post-treatment, and in the follow-up period (at 3 and 6 months and at 1 year of follow-up from the end of the treatment program). The assessment was carried out by a researcher who was blind to the patient allocation process. The evaluators of two participating centers were trained to use scales for inter-rater reliability by rating each of the scales with practical cases. The therapists from both centers received a face-to-face training session and were provided with the same materials that would later be offered to the patients in the intervention program. 

### 2.4. Measures 

The assessment protocol is widely described in a previous article by González-Ortega et al. [34]. Sociodemographic variables (age, gender, educational level, socioeconomic level, employment status, family history of psychiatric disorders) were collected using a clinical interview at baseline. Clinical and cannabis/other substance use-related variables were assessed at baseline, post-treatment, and in the follow-up (3, 6, and 12 months of follow-up from the end of the intervention program). 

#### 2.4.1. Primary Outcome

The primary outcome was the reduction in cannabis use severity measured by Europ-ASI [36,37], (which assesses the severity of the substance use problem) and DSM-IVTR criteria. 

#### 2.4.2. Secondary Outcomes

##### Cannabis Use

Cannabis and other substance use, including the frequency of use (daily, weekend, weekly, monthly), the amount of cannabis use, the age of onset of use, the history of use (years), and urine samples were collected. 

Cannabis use was monitored by patient reports and with repeated urine toxicology tests. Toxins in urine analysis were performed with an immunochromatographic test to detect qualitatively drug metabolites at baseline, at sessions 4 and 8 of the psychological treatment, at post-treatment, and at 3 and 6 months and 1 year of follow-up. 

##### Clinical Variables and Functioning

Patients were diagnosed according to the DSM-IV-TR criteria using the Structured Clinical Interview for the Diagnostic and Statistical Manual of Mental Disorders, Axis I Disorders (SCID-I) [38]. 

The Clinical Global Impression Scale (CGI) [39] was used to assess symptom severity (CGI-Severity) and global improvement (CGI-Improvement). 

The illness awareness of patients was measured using the scale to assess Unawareness in Mental Disorders (SUMD) [40,41] and medication adherence was assessed with the 4-item Morisky Medication Adherence Scale (MMAS) [42,43].

Positive, negative, and general psychotic symptoms were measured using the Positive and Negative Syndrome Scale (PANSS) [44,45]. 

Depressive symptoms were assessed with the Hamilton Depression Rating Scale (HDRS) [46,47] and anxiety symptoms with the Hamilton Anxiety Scale (HAM-A) [48,49].

The Young Mania Rating Scale (YMRS) [50,51] was used to measure the manic symptoms. 

The functioning of patients was measured using the Functioning Assessment Short Test (FAST) [52], which assesses six specific areas of functioning: autonomy, occupational functioning, cognitive functioning, financial issues, interpersonal relationships, and leisure time. 

##### Determination of Treatment Response 

The efficacy of the therapy was assessed analyzing the treatment response of patients. The treatment response in relation to the cannabis cessation was determined by comparing the time it took for patients in both groups to decrease the severity of consumption, evaluated through the decrease in the Europ-ASI scale score (one category of severity) from baseline to one year of follow-up. 

The clinical response was defined as a significant improvement in the psychopathology, assessed by a decrease in symptoms from baseline to endpoint of follow-up. The primary efficacy measure was the treatment response, defined according to other studies, as at least a 20% reduction in PANSS total score from baseline to the endpoint [53]. Secondary efficacy variables included the response according to a decrease in the total score ≥ 50% in HDRS [54], HAM-A [55], and YMRS [56] scales. 

The functional response was defined as the reduction of one category of severity of functional impairment, according to the cut-off values of the FAST scale [57], which has been validated in this population [58,59]. 

### 2.5. Intervention Programs

Patients were randomized into two treatment groups (TAU or CBT-CC). 

#### 2.5.1. Treatment as Usual (TAU) 

TAU refers to the combined clinical treatment provided to FEP patients that included the pharmacological treatment prescribed by the psychiatrist and an individual psychological therapy involving psychoeducation and CBT, following the same format as the CBT-CC group, that is, 1-h sessions once a week for 16 weeks. 

Therapy sessions include career counseling and information to enable patients to understand and be able to manage their disease, providing them cognitive–behavioral tools for symptom management and relapses and to contribute to their well-being. 

The first part of the psychological program (sessions 1–9) was composed of psychoeducational sessions aimed at improving patients’ insight into their illness, treatment adherence, prodromal identification, early intervention to prevent relapses, and a healthy lifestyle. The second part of the intervention (sessions 10–16) included cognitive–behavioral techniques for symptom and thought management (anxiety management techniques) and social and problem-solving skills. 

#### 2.5.2. Specific CBT for Cannabis Cessation (CBT-CC)

The CBT-CC group received a specific individual CBT for cannabis cessation composed of 1-h sessions once a week for 16 weeks, in addition to the pharmacological treatment prescribed by the psychiatrist. 

The treatment included a cognitive–behavioral approach that integrated three fundamental aspects: (1) motivational strategies to develop a good therapeutic alliance and motivation for change, (2) cognitive–behavioral techniques for cannabis abstinence, symptom management, and improvement in psychosocial functioning, and (3) a specific intervention for change maintenance and relapse prevention. The content of the sessions is as follows:

Sessions 1–4: The first four sessions involved motivational interviewing [60], followed by brief psychoeducation focused on general information about cannabis and psychosis: (a) psychosis and substance use, (b) medication and treatment adherence, (c) awareness of the vulnerability, (d) recognition of symptoms, (e) healthy lifestyle, and (f) risk and protective factors.

Sessions 5–14: The second part of the program was focused on commitment to change [61] and included the following aspects:

Behavioral therapy:

- Anxiety management techniques;
- Stimulus control;
- In vivo exposure therapy with response prevention, identifying triggers and beliefs that could lead to substance use and exacerbation of psychotic symptoms and exposure to such triggers.

Cognitive therapy:

- Specific techniques for managing thoughts about the consumption and use of cannabis and other substances (craving/abstinence) and symptom management;
- Cognitive restructuring; identifying and refuting cognitive distortions;
- Training in problem-solving;
- Training in social skills; assertiveness; skills to refuse drugs and changes in lifestyle.

Sessions 15–16: The third part of the program included a specific intervention for relapse prevention, focused on the identification of high-risk situations that could lead to maintenance of substance use and increased severity and chronicity of psychotic symptoms, as well as the teaching of coping skills for such situations. 

### 2.6. Statistical Analysis 

SPSS Statistics for Windows (version 23.0, IBM, Armonk, NY, USA) was used for the statistical analysis, with the significance level set at *p* ≤ 0.05.

Differences in baseline sociodemographic and clinical characteristics between intervention groups were analyzed using the χ^2^ test for categorical variables and Student’s *t*-test for continuous variables.

With respect to cannabis consumption, the concordance between results identified in urine toxicology tests and clinician rating scale determined by participant-reported cannabis use and DSM-IVTR [35]/Europ-ASI [36,37] criteria was evaluated using the Kappa Coefficient. 

Ordinal mixed-effects models were used to analyze the differences in cannabis use reduction over time (from baseline to the 12 months of follow-up) between the two intervention groups. The efficacy of the therapy for cannabis cessation and the effect of cannabis abstinence/reduction in terms of clinical and functional outcomes for patients were analyzed with lineal mixed-effects models for the analyses of repeated measures. First, we assessed the individual effect of gender, age, civil status, socioeconomic level, educational level, family history, adherence to treatment, age at onset of cannabis use, and other substance use. Those variables for which the ANOVA test had a *p*-value < 0.1 were included in the final model. Finally, multivariate mixed-effects models were fitted, including the intervention group (CBT-CC vs. TAU) or the cannabis abstinence/reduction vs. continued use group, those confounding variables previously selected, and the time variable. The analysis of cannabis abstinence/reduction vs. continued use was performed with all samples and the effect of the intervention group was controlled. The reference category for the grouping variables was the TAU group and the continued cannabis use, respectively. A random effect was included in these models to account for the repeated measure structure of the data and was performed with maximum likelihood methods.

Survival analysis (Kaplan–Meier and adjusted Cox regression) was used to determine differences between intervention groups in terms of time to treatment response. 

## 3. Results

### 3.1. Baseline Characteristics of the Sample 

A total of 69 FEP patients (35 for the CBT-CC group and 34 for the TAU group) met inclusion criteria, consented to participate, and were initially included in the study. Of these, four (three in the CBT-CC group and one in the TAU group) dropped out or did not complete all intervention sessions. The analyses were performed with patients who completed all treatment sessions, and performed at least the post-treatment assessment, so the final study sample consisted of 65 patients (34 were assigned in the CBT-CC group and 31 in the TAU group) (Figure 1). 

The majority of the sample (72.3%) were men and (27.7%) were women. The mean age of the sample was 25.78 years (7.08), and the age of cannabis onset was 15.55 (2.55). Thirty-nine (60%) of patients met the criteria for cannabis abuse and 26 (40%) for cannabis dependence. 

There were no statistically significant differences between the two treatment groups on any baseline measures. The sociodemographic, clinical, and cannabis use baseline characteristics of the participants of the two groups are presented in Table 2. 

### 3.2. Outcomes 

#### 3.2.1. Primary Outcome: Evolution of Cannabis Use Severity over 12 Months

When cannabis use of the CBT-CC and TAU groups was compared over time (from baseline to the 12 months of follow-up), the ordinal mixed models’ results revealed significant differences between the two groups regarding the change in cannabis use, assessed by Europ-ASI scores. The CBT-CC group had a greater decrease in the severity of consumption compared to the TAU group (β = 0.190; *p* < 0.001). Patients who received specific therapy for cannabis use cessation had a greater reduction in the cannabis use severity than TAU at post-treatment (β = −1.418, *p* < 0.001) and in all follow-up visits (3 months: β = −0.990, *p* = 0.002; 6 months: β = −1.167, *p* ≤ 0.001; 12 months: β = −1.091, *p* = 0.004).

Specifically, 50% of the CBT-CC group sample achieved abstinence at post-treatment versus 12.9% of TAU, and the severity of their cannabis use was also lower in the follow-up compared to TAU (χ^2^ = 29.055; *p* < 0.001). Namely, 29.4% of patients used, 17.6 abused, and only 2.9% met the criteria for cannabis dependence, whereas the majority of TAU group patients were dependent (48.4%) on or abused (35.5%) cannabis. These results were maintained at follow-up, with a higher percentage of abstinence or reduction in the severity of consumption in patients of the CBT-CC group compared to TAU (Table 3).

**Figure 2:**
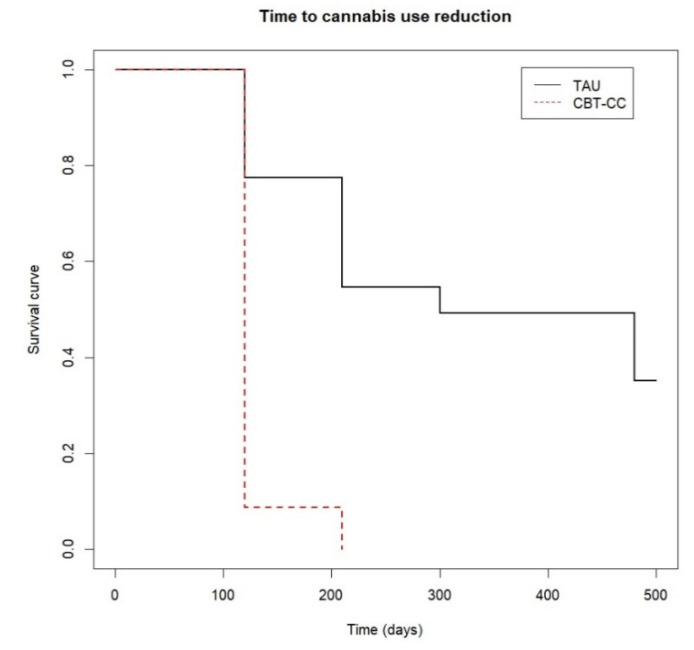
Time to cannabis use reduction.

The survival analysis also showed that the CBT-CC group achieved a decrease in cannabis use much earlier than the TAU group (log-rank test: χ^2^ = 34.34, *p* ≤ 0.001). In fact, the CBT-CC group had seven times more likely to decrease a category of the severity of consumption than the TAU group (HR = 7.681, 95% CI: 3.58, 16.48). Specifically, the median time indicated that 50% of the CBT-CC group patients reduced cannabis use in less than 120 days, (which coincided with the end of therapy), while in the TAU group the median time was 300 days (Figure 2). 

There was a high concordance (Kappa index) between cannabis use identified in urine toxicology tests and clinician rating scale determined by participant-reported cannabis use and Europ-ASI/DSM-IVTR criteria, at post-treatment (k = 0.893) and in each follow-up visit (3 months: k = 1; 6 months: k = 0.941; 12 months: k = 0.924, *p* < 0.001). 

#### 3.2.2. Secondary Outcomes

##### Frequency and Amount of Cannabis Use Thought the Follow-Up 

At baseline, patients in both groups consumed cannabis daily and there were no differences in the amount of cannabis consumed, with an average of 2.204 (0.956) grams per day in the TAU group and a mean of 1.883 (0.956) grams in the CBT-CC group. However, there were statistically significant differences between two groups at post-treatment and in the follow-up, with a greater reduction in the amount and frequency of cannabis used in the CBT-CC group compared to TAU. Forty-seven percent of the CBT-CC group patients were abstinent post-treatment and more than half remained abstinent at 3, 6, and 12 months of follow-up. However, only 12.9% of TAU group patients discontinued cannabis post-treatment and the majority of them continued to use cannabis daily at follow-up (Table 4).

##### Clinical and Functional Outcomes

Regarding clinical and functional outcomes of patients, although both groups improved their outcomes throughout the follow-up, the experimental group obtained a greater reduction in psychotic symptoms over time (except at the 12-month follow-up visit) than the control group (post-treatment: β = −5.225, *p* < 0.001; 3 months: β = −4.143, *p* = 0.007; 6 months: β = −5.045, *p* = 0.003). The CBT-CC group also showed a greater improvement in functional outcome (β =−8.111; *p* = 0.036) compared to the TAU group at post-treatment, with no significant differences between groups in the follow-up. There were no differences between groups in other clinical outcomes evolution (negative and general psychotic symptoms, depressive, manic, anxiety symptoms, or awareness of disease) over the 12 months of follow-up. 

**Figure 3:**
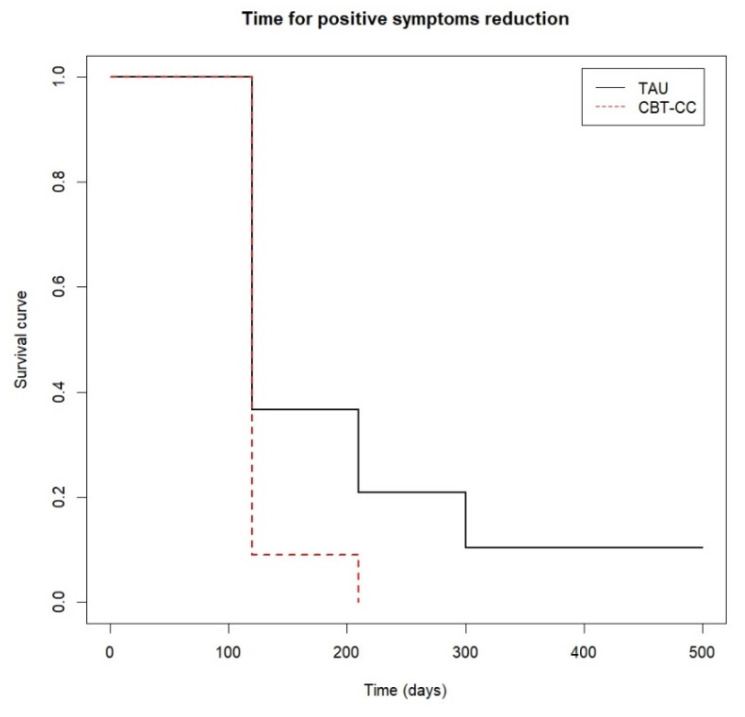
Time to positive psychotic symptoms reduction.

**Figure 4:**
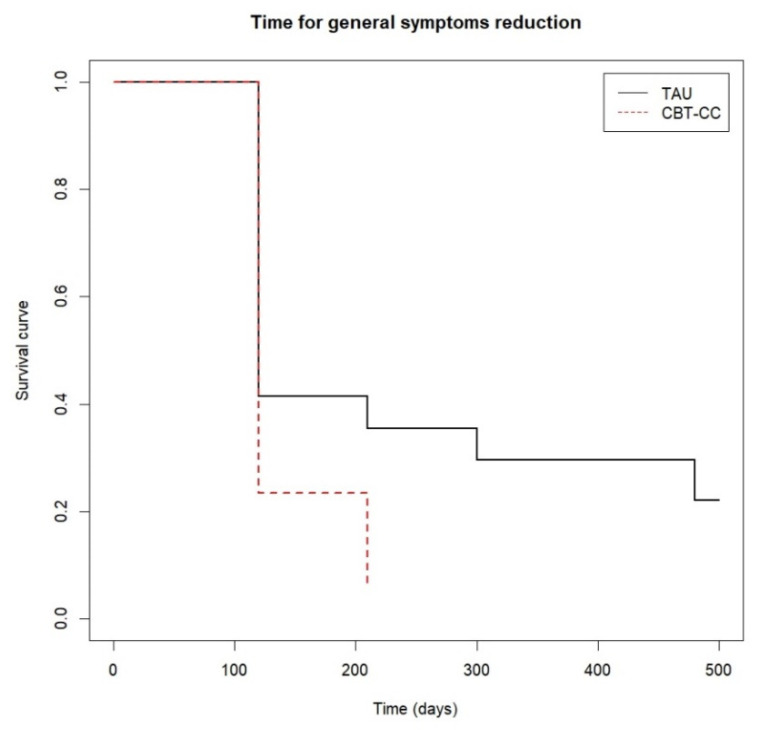
Time to general psychotic symptoms reduction.

**Figure 5:**
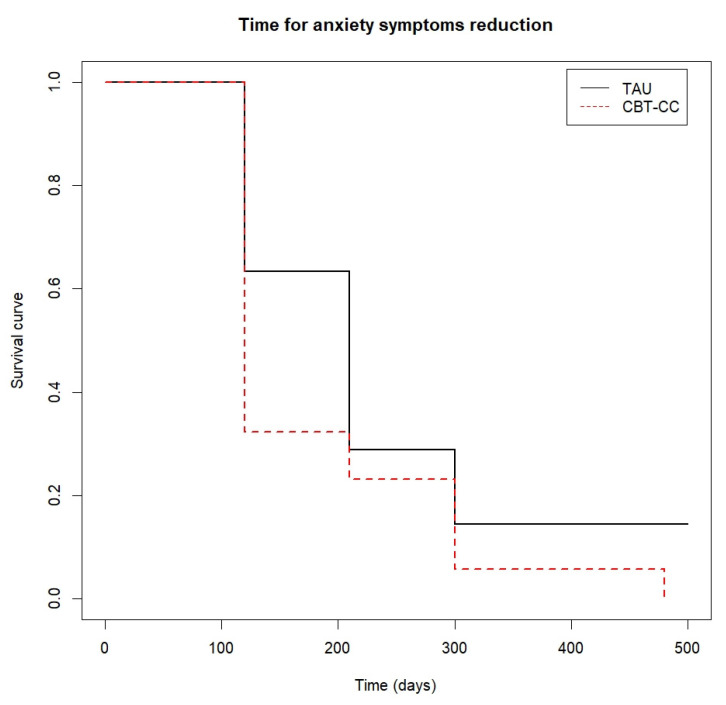
Time to anxiety symptoms reduction.

**Figure 6:**
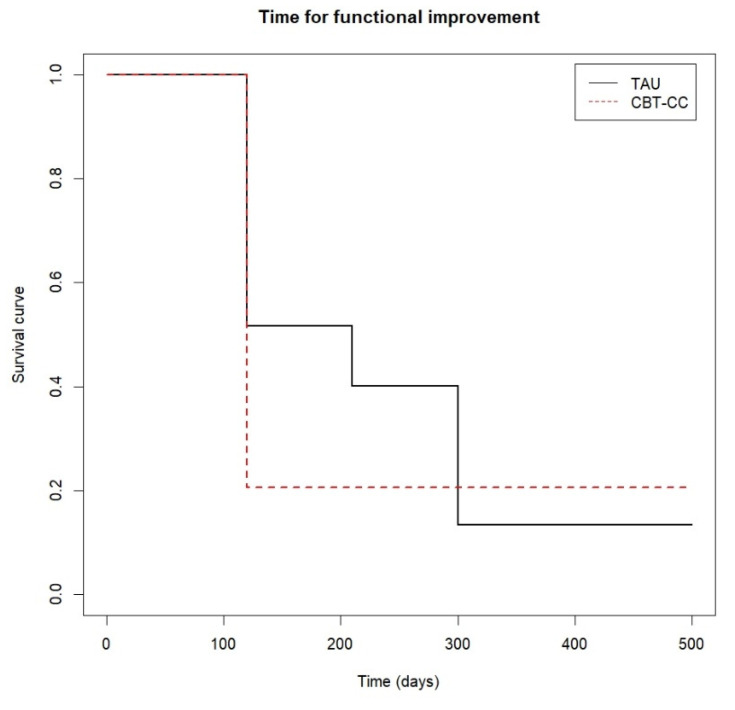
Time to functional improvement.

When we analyzed the survival analysis of the time it took each group to reach the clinical improvement criterion, the CBT-CC group showed a greater acceleration in the achievement of the improvement criterion, according to the decrease in the score of the PANSS P and G, HAM-A and FAST scales, compared to the TAU group (Figure 3, Figure 4, Figure 5 and Figure 6). Regarding positive symptoms, Figure 3 shows that the CBT-CC group achieved the reduction earlier than the TAU group (log-rank test: χ^2^ = 15.78, *p* = 0.003). In fact, 90% of CBT-CC group patients achieved a response at post-treatment (120 days), while the TAU group needed 6 months (300 days). The CBT-CC experimental group was twice as likely to reduce positive symptoms by at least 20%, compared to the TAU group (HR = 2.148, 95%CI: 1.209, 3.817).

There were significant differences between the curves of both groups at follow-up (log-rank test: χ^2^ = 8.87, *p* = 0.010). Approximately 80% of the CBT-CC group achieved the response criterion post-treatment, while the 80% of the TAU group decreased the general psychotic symptoms at 12 months of follow-up, with a greater probability of reducing symptoms in the CBT-CC group in the follow-up (HR = 1.74, 95%CI: 0.963, 3.162) (Figure 4). 

The CBT-CC group was also more likely to achieve a 50% reduction in anxiety score (log-rank test: χ^2^ = 3.85, *p* = 0.05), being the median time in this group of 120 days versus 210 days in the TAU group (HR = 1.775, 95%CI: 0.993, 3.172) (Figure 5).

Finally, Figure 6 shows that approximately 80% of the CBT-CC group patients managed to reduce one category in the FAST scale at 120 days (post-treatment) from baseline, while in the TAU group at 210 days (3 months) only 60% of patients achieved improvement criteria. That is, the CBT-CC group managed to improve functionality in less time than the TAU group (log-rank test: χ^2^ = 7.68, *p* = 0.05, HR = 1.686, 95%CI: 0.922, 3.081).

##### Relationship between Cannabis Cessation/Reduction and Clinical and Functional Outcomes

The relation between cannabis cessation and/or reduction and clinical and functional outcomes of patients was analyzed, and the results showed that patients who stopped or reduced cannabis use during the follow-up, decreased general psychotic (β = −0.754; *p* = 0.022) compared to those who continued to cannabis use. In addition, patients who stopped and/or reduced their consumption had a better awareness of the disease than those who continued to consume during follow-up (β = 0.197; *p* = 0.050). 

## 4. Discussion

This randomized controlled trial aimed to compare the efficacy of a specific CBT program for cannabis use cessation with standard treatment in patients with FEP cannabis users. 

The primary outcome of the study was that CBT-CC patients had a greater decrease in the severity of cannabis use over time, compared to the TAU group. Moreover, the treatment response was faster in the CBT-CC group, reducing their cannabis use in around 120 days, (which coincided with post-treatment), while in the control group the time to reduce the consumption was 300 days (around 6 months of follow-up). Very few RCTs have evaluated psychosocial interventions for cannabis use cessation in FEP, and these have not found benefits in terms of reduction in cannabis use in the follow-up, compared with TAU [27,28,29,30,31,32]. Therefore, our results are promising and suggest that our intervention could be useful for cannabis cessation in FEP patients. 

The second main finding of the study is that our psychological therapy also proved to be effective in improving clinical and functional outcomes of FEP. The CBT-CC group had a greater reduction in positive psychotic symptoms and a greater improvement in functional outcomes post-treatment compared to the TAU group. Moreover, the CBT-CC group reached the treatment response, that is, the clinical and functional improvement criterion, faster in the follow-up than the TAU group. Specifically, the majority of patients (80–90%) who received CBT-CC reduced the positive and general psychotic symptoms earlier (at post-treatment), while the TAU group needed between 6 and 12 months to reach the same percentages of response. In addition, the CBT-CC group also decreased anxiety symptoms and achieved functional response in less time (at post-treatment versus 3 and 6 months, respectively). These results have not been tested in other RCTs that have assessed the efficacy of an integrated psychological intervention to reduce cannabis use and improve the outcome of FEP [27,29,31,32]. Only in the study conducted by Madigan et al. [30], the intervention improved the quality of life of patients at 3 months and one-year follow-up; however, this was not associated with a reduction in cannabis use or improvement in clinical outcomes. Our findings revealed that the success of therapy in symptom improvement may be related to the dual therapeutic approach, aimed at both cannabis cessation and symptom improvement so that the cannabis reduction/cessation enhanced the efficacy of strategies aimed at managing symptoms.

The third finding of the study was that cannabis cessation influenced the clinical and functional outcomes of FEP. Patients who stopped and/or reduced cannabis use during the follow-up decreased psychotic symptoms and had a better awareness of the disease compared to those who continued cannabis use during follow-up. Other observational longitudinal studies confirm that cannabis use cessation has been related to an improvement in clinical and functional outcomes of FEP in the follow-up [18,20]. In a recent study, Setién-Suero et al. [26] also found that persistent cannabis FEP users had more severe symptoms and poorer functionality compared to ex-users and never-users at a 10-year follow-up. Moreover, patients who stopped cannabis do not differ from those who had never consumed. 

Our findings have several strengths and relevant implications for clinical practice and underscore the importance of treating early FEP cannabis users in order to improve the prognosis of their disease. Since cannabis has a significant role in the prognosis of FEP patients and given that the deleterious effect of cannabis could be reversed with the decrease or cessation of consumption, there is a need for early intervention for cannabis cessation after the onset of FEP. Although CBT has previously proven to be a useful therapeutic approach to treat and improve clinical and functional outcomes in FEP [62], to date no specific therapies had been found that have been shown to be effective for FEP cannabis users. This integrated psychological CBT intervention has been shown to be effective both in cannabis cessation use and in improving clinical and functional outcomes of FEP cannabis users, following the paradigm of precision psychotherapy [63]. 

The key aspects of the therapy are developing a good therapeutic alliance and motivation for change, training in coping skills and self-management strategies, and relapse prevention. Our results support the importance of developing a good therapeutic alliance in the first therapy sessions, to facilitate motivation for change in this type of patient, who often have little illness awareness, and minimize the consequences of cannabis use [64]. A crucial psychological treatment target is to increase awareness about the problematic cannabis use behavior and the impact of cannabis use on the prognosis of the psychosis. The objective is to know the cannabis use behavior, the perceptions, and knowledge about cannabis use as well as provide them with a brief psychoeducation focused on general information about cannabis use, psychosis, and the relationship between cannabis use and psychosis. It is also essential to establish a change plan with the patient [60,61], considering not only abstinence but also the reduction in cannabis use severity for those patients who are not ready to reduce or quit cannabis, as a change goal and therapeutic objective [65]. The strategies aimed at improving the awareness of the adverse impact of cannabis use are critical to cannabis cessation and maintenance of change [66] (Hides et al., 2016). In our FEP cannabis users’ sample, patients who stopped and/or reduced their consumption, had a better awareness of the disease compared to those who continued to consume during follow-up, so increased awareness of cannabis use may be critical to increasing motivation for cannabis cessation and strengthen the commitment to change and ultimately, improve clinical and functional outcomes.

In the second phase of treatment, the aim was to help the patient make the proposed changes through training in coping skills and self-control strategies, applying specific cognitive–behavioral techniques to treat both the psychotic disorder and cannabis abstinence. Our findings showed that psychological therapy, with a dual approach aimed at both cannabis cessation and psychosis, proved to be effective in both reducing cannabis and improving psychotic symptoms and functional outcomes. Since cannabis use has been related to higher severity of positive psychotic symptoms (11, 18, 19) and a worse prognosis in patients with FEP (20–22), it is essential to teach the patient coping strategies such as cognitive distraction or cognitive restructuring techniques to learn to manage positive symptoms and reduce the likelihood of relapse. Likewise, providing the patient with strategies to deal with both situations of risk of consumption (training in skills to reject cannabis use) and situations of daily life (communication skills, assertiveness training, skills to improve social cognition), will improve patient functioning and the course of the disease.

Finally, it is common that during the recovery process relapses occur, so a key aspect of our therapy is to develop strategies for change maintenance and relapse prevention [67]. In our FEP sample, the CBT-CC group achieved a significant decrease in the severity of cannabis use post-treatment and it was maintained at follow-up. The treatment response was also reached post-treatment and was maintained over time, reinforcing the importance of developing a relapse plan.

This study has some limitations. One of the limitations of the study is related to the usual loss of patients in the follow-up in this type of clinical trial with a longitudinal design, which should be taken into consideration in interpreting the efficacy of the therapy. In future studies, it is recommended to develop motivational strategies for patient retention in follow-up evaluations. Our study had a follow-up of 1 year; a longer follow-up period should be considered in future research. Another limitation is that the psychological treatment programs had an individual format, while others employed a group or family approach, given the relevance of the family environment [68]. Finally, the influence of the type of cannabis used (marijuana, hashish) and/or the form of consumption (smoked or inhaled) on the prognosis of FEP could be considered in future studies. 

## 5. Conclusions

The results of this study suggest that early intervention based on a specific CBT program for cannabis cessation, that combines therapeutic strategies aimed at addressing both mental and addictive disorders for patients with FEP and cannabis use comorbidity, may be effective in reducing the cannabis use severity, in addition to improving clinical and functional outcomes of FEP patients. 

## Figures and Tables

**Table 1:** Classification of cannabis use for selection of participants.

Severity of Consumption	DSM-IV-TR ^a^ Criteria for Abuse or Dependence	Europ-ASI ^b^ Scores
Dependence	Meet at least minimal DSM-IV-TR criteria for cannabis dependence	8–9
Abuse	Meet ≥ 1 DSM-IV-TR criteria for cannabis abuse	4–7
Use	Meet DSM-IV-TR criteria for cannabis abuse but not the duration criterion (≥12 months) or ≥12 months of use but do not meet any DSM-IV-TR criteria for cannabis abuse	2–3
No use	No significant symptoms	0–1

^a^ Revised Fourth Edition of the Diagnostic and Statistical Manual of Mental Disorders (DSM-IV-TR) [35]. ^b^ European Addiction Severity Index (Europ-ASI) [36,37].

**Table 2:** Baseline sociodemographic and clinical characteristics of the sample.

		TAU (*n* = 31)	CBT-CC (*n* = 34)	t/χ^2^
Gender	Male	24 (77.4%)	23 (67.6%)	χ^2^ = 0.773, *p* = 0.379
Age		27.19 (7.21)	24.50 (6.81)	t = 1.548, *p* = 0.127
Marital status	Single	29 (93.5%)	33 (97.1%)	χ^2^ = 3.126, *p* = 0.209
	Married	2 (6.5%)	0 (0%)	
	Others	0 (0%)	1 (2.9%)	
Socioeconomic level	Low	9 (29.0%)	8 (23.5%)	χ^2^ = 0.392, *p* = 0.822
	Medium	15 (48.4%)	19 (55.9%)	
	High	7 (22.6%)	7 (20.6%)	
Educational level	Primary	10 (32.3%)	15 (44.1%)	χ^2^ = 2.182, *p* = 0.336
	Secondary	16 (51.6%)	17 (50.0%)	
	College	5 (16.1%)	2 (5.9%)	
Adherence	Bad	16 (53.3%)	19 (57.6%)	χ^2^ = 0.115, *p* = 0.735
	Good	14 (46.7%)	14 (42.4%)	
Family history	No	15 (51.7%)	20 (55.6%)	χ^2^ = 0.195, *p* = 0.659
	Yes	14 (48.3%)	16 (44.4%)	
Age of cannabis onset		15.87 (2.87)	15.26 (2.22)	t = 0.957, *p* = 0.342
Years of cannabis use		8.91 (6.55)	11.10 (6.49)	t = 1.349, *p* = 0.182
Cannabis	Abuse	17 (54.8%)	22 (64.7%)	χ^2^ = 1.967, *p* = 0.374
	Dependence	14 (45.2%)	12 (35.3%)	
Tobacco	Use	4 (12.9%)	2 (5.9%)	χ^2^ = 3.607, *p* = 0.307
	Abuse	0 (0%)	2 (5.9%)	
	Dependence	27 (87.1%)	29 (85.3%)	
Alcohol	Use	17 (54.8%)	15 (44.1%)	χ^2^ = 4.319, *p* = 0.229
	Abuse	6 (19.4%)	9 (26.5%)	
	Dependence	4 (12.9%)	1 (2.9%)	
Cocaine	Use	0 (0%)	2 (5.9%)	χ^2^ = 2.066, *p* = 0.559
	Abuse	2 (6.5%)	3 (8.8%)	
	Dependence	2 (6.5%)	2 (5.9%)	
Amphetamines	Use	4 (12.9%)	3 (8.8%)	χ^2^ = 0.760, *p* = 0.859
	Abuse	2 (6.5%)	4 (11.8%)	
	Dependence	3 (9.7%)	3 (8.8%)	
Other substances	Use	1 (3.2%)	2 (5.9%)	χ^2^ = 1.345, *p* = 0.510
	Abuse	0 (0%)	0 (0%)	
	Dependence	1 (3.2%)	0 (0%)	
Treatment	Antipsychotics	28 (90.3%)	33 (97.1%)	χ^2^ = 2.272, *p* = 0.132
	Antidepressants	3 (9.7%)	2 (5.9%)	χ^2^ = 0.334, *p* = 0.563
	Mood stabilizers	3 (9.7%)	5 (14.7%)	χ^2^ = 0.376, *p* = 0.540
	Benzodiazepines	17 (54.8%)	21 (61.8%)	χ^2^ = 0.319, *p* = 0.572
PANSS P		16.23 (5.93)	18.59 (4.68)	t = −1.791, *p* = 0.078
PANSS N		16.52 (8.43)	14.73 (7.18)	t = 0.916, *p* = 0.363
PANSS G		33.13 (8.29)	30.32 (4.83)	t = 1.646, *p* = 0.106
HDRS		14.19 (5.78)	11.88 (6.01)	t = 1.576, *p* = 0.120
HAM-A		7.97 (5.23)	7.94 (4.13)	t = −0.030, *p* = 0.976
YMRS		8.94 (10.18)	7.79 (9.94)	t = 0.457, *p* = 0.649
SUMD		9.10 (3.02)	9.03 (3.97)	t = 0.077, *p* = 0.939
FAST		37.32 (10.52)	34.32 (12.70)	t = 1.031, *p* = 0.306

**Table 3:** Cannabis use severity at follow-up.

	Post		3 months		6 months		12 months	
	*n* (%)	χ^2^/*p*	*n* (%)	χ^2^/*p*	*n* (%)	χ^2^/*p*	*n* (%)	χ^2^/*p*
TAU								
No	4 (12.9%)		4 (22.2%)		4 (26.7%)		2 (20.0%)	
Use	1 (3.2%)		3 (16.7%)		0 (0%)		2 (20.0%)	
Abuse	11 (35.5%)		6 (33.3%)		6 (40.0%)		3 (30.0%)	
Dependence	15 (48.4%)	χ^2^ = 29.055, *p* < 0.001	5 (27.8%)	χ^2^ = 15.697, *p* = 0.001	5 (33.3%)	χ^2^ = 16.385, *p* = 0.001	3 (30.0%)	χ^2^ = 6.025, *p* = 0.10
CBT-CC								
No	17 (50%)		15 (68.2%)		13 (65.0%)		10 (55.6%)	
Use	10 (29.4%)		6 (27.3%)		5 (25.0%)		5 (27.8%)	
Abuse	6 (17.6%)		1 (4.5%)		2 (10%)		2 (11.1%)	
Dependence	1 (2.9%)		0 (0%)		0 (0%)		1 (5.6%)	

**Table 4:** Frequency and amount of cannabis use throughout the follow-up.

	TAU		CBT-CC			
	Mean/*n*	SD/%	Mean/*n*	SD/%	t/χ^2^	*p*
Amount						
Post-treatment	1.725	1.120	0.612	0.776	4.693	<0.001
3 months	1.165	0.927	0.346	0.622	3.250	0.003
6 months	1.188	1.004	0.299	0.442	3.216	0.005
12 months	1.188	0.883	0.479	0.540	2.735	0.011
Frequency						
Post-treatment						
No	4	12.9%	16	47.1%	18.871	<0.001
Daily	27	87.1%	12	35.3%		
Weekend	0	0%	2	5.9%		
Weekly	0	0%	4	11.8%		
3 months						
No	4	23.5%	14	66.7	8.811	0.012
Daily	13	76.5%	6	28.6%		
Weekly	0	0%	1	4.8%		
6 months						
No	4	26.7%	14	66.7%	11.131	0.011
Daily	11	73.3%	4	19%		
Weekend	0	0%	2	9.5%		
Weekly	0	0%	1	4.8%		
12 months						
No	2	20%	9	45%	4.964	0.291
Daily	6	60%	5	25%		
Weekend	0	0%	1	5%		
Weekly	2	20%	3	15%		
Monthly	0	0%	2	10%		

## Data Availability

The data are contained within the article.
